# Interferon gamma expression and mortality in unselected cohorts of urothelial bladder cancer patients

**DOI:** 10.1371/journal.pone.0271339

**Published:** 2022-08-30

**Authors:** Christina Gillezeau, Naimisha Movva, Maaike van Gerwen, Karma Rabon-Stith, Norah Shire, Philip Zachary Brohawn, Emanuela Taioli, Jon Fryzek

**Affiliations:** 1 Institute for Translational Epidemiology at the Icahn School of Medicine at Mount Sinai, New York, New York, United States of America; 2 EpidStrategies, Rockville, Maryland, United States of America; 3 Department of Otolaryngology- Head and Neck Surgery, Icahn School of Medicine at Mount Sinai, New York, NY, United States of America; 4 AstraZeneca, Gaithersburg, Maryland, United States of America; Seoul National University Hospital, REPUBLIC OF KOREA

## Abstract

**Background:**

The role of interferon gamma (IFN-γ) expression in long-term survival has not been studied in patients with urinary bladder cancer (UBC). IFN-γ expression was characterized among various UBC patient cohorts to assess if IFN-γ status is associated with overall survival (OS).

**Methods:**

A tumor-based IFN-γ gene signature was evaluated among adult UBC patients newly diagnosed between 2004 and 2017 from two hospital systems in New York. Patient cohorts included metastatic (stage IV or progressing to stage IV [MBC]), muscle-invasive (stages T2a to T4a [MIBC]), and non–muscle-invasive (carcinoma *in situ* or stages 0a, 0is, and I [NMIBC]) disease. Descriptive analyses were conducted comparing IFN-γ signature in the highest tertile to those in the lowest two tertiles.

**Results:**

234 patients with bladder cancer were evaluated (56 MBC, 38 MIBC, and 140 NMIBC). Median OS was only reached in the MIBC cohort for those with an IFN-γ signature in the lowest two tertiles (15.03 months [95% CI, 8.50–50.60]). Those with an IFN-γ signature in the highest tertile had a decreased risk of mortality in all cohorts indicating better survival, but this was statistically significant in only the MIBC cohort (adjusted HR = 0.09 [95% CI, 0.01–0.73]).

**Conclusion:**

IFN-γ signature status was associated with a decreased mortality risk in all cohorts, particularly MIBC, indicating that it may be a prognostic marker of survival in patients with UBC.

## Introduction

Urinary bladder cancer (UBC) is the ninth most common cancer worldwide, with nearly 500,000 new cases diagnosed annually [1]. UBC is typically categorized by the level of invasiveness of the tumor into one of three groups based on the TNM staging system: non-muscle invasive bladder cancer (NMIBC), which includes Tis-T1 lesions, muscle invasive bladder cancer (MIBC), which includes T2-T3 lesions, or metastatic bladder cancer (MBC) [2].

Conservative treatment for NMIBC generally includes a partial or full transurethral resection of the bladder tumor in combination with a perioperative intravesical chemotherapy [3–5]. NMIBC lesions are typically also treated with immunotherapy, the most common of which is bacillus Calmette-Guérin (BCG) therapy [3, 4, 6, 7]. The role of immunotherapy in bladder cancer was first demonstrated by the success of BCG instillation in localized bladder cancer [8], where it was shown to reduce the rates of recurrence and progression by up to 40% [6, 7]. This success encouraged the investigation into the role of BCG and other immunotherapy agents in the treatment of MIBC as well, but a deeper understanding of the mechanism of immunotherapy in UBC and, in particular, the ability to predict which tumors may be responsive, is essential. For patients with UBC who have a minimal risk of progression or recurrence, conservative management is preferred to maintain a functional bladder and increase patient quality of life [8].

It has been hypothesized that higher levels of interferon gamma (IFN-γ) mRNA signature expression may predict increased immunotherapy efficacy [9], which in turn is associated with enhanced T-cell mediated antitumor immune response and a better prognosis [10]. This hypothesis is consistent with the postulated mechanism of action of immunotherapies in other cancers including non-small cell lung cancer [11], melanoma, squamous cell carcinomas of the head and neck, and gastric cancers [9, 12–19]. It is believed that IFN-γ expression in patients with UBC may also therefore be predictive of tumor aggressiveness, treatment response, and long-term patient outcomes; however, data are currently limited.

This study determined the IFN-γ signatures of tumors from patients with UBC and investigated the association between the IFN-γ signatures and survival among various UBC patient cohorts (NMIBC, MIBC, and MBC).

## Methods

### Study population

In this retrospective observational study, available tissue or biopsy samples of newly diagnosed patients with urothelial bladder cancer were collected from North Shore Long Island Jewish Health System (*n* = 120; 43%) or Mount Sinai Health System (*n* = 162; 57%), both located in New York, New York. Cohorts of patients with NMIBC (*n* = 163), MIBC (*n* = 51), or MBC (*n* = 68) were assembled. Electronic medical records (EMR) were reviewed to gather detailed demographic and clinical information. Institutional Review Board (IRB) approvals from both institutions were obtained (Northwell Health IRB: IRB 08-094A; Mount Sinai IRB: HS 15–00495). Informed patient consent was waived by the IRBs due to the retrospective nature of the study.

### Study eligibility

Patients were 18 years or older at diagnosis and diagnosed with UBC between 2004 and 2017 to be eligible for inclusion. Samples were collected for diagnostic purposes. For the MBC cohort, patients with a histologically confirmed diagnosis of stage IV or progressing to stage IV bladder cancer were included. The MIBC cohort comprised patients with a histologically confirmed diagnosis of stage T2a, T2b, T3a, T3b, or T4a bladder cancer. In the NMIBC cohort, patients had a histologically confirmed diagnosis of stage Ta, T1, or Tis (carcinoma *in situ*) bladder cancer or stage 0a, 0is, and I bladder cancers who received five of six BCG instillations. Transitional or mixed histologies were accepted. Further inclusion criteria for the study were the availability of 1) sufficient cancer tissue or biopsy sample to provide two curls of 20 μm per patient and 2) detailed information on demographics, pathology, treatment, and clinical outcomes, including treatment and follow-up information to determine overall survival (OS) and progression-free survival (PFS).

### Variables

**Fig 1:**
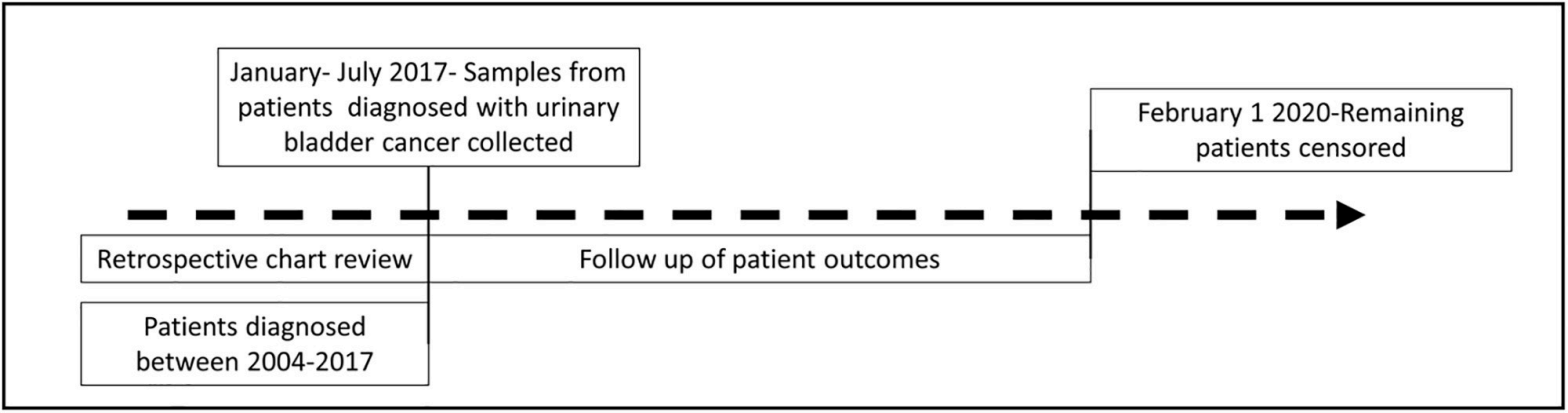
Study flow diagram. Urinary bladder cancer study. Mt Sinai hospital system and North Shore Long Island Jewish health system, New York, New York. 2004–2017.

Gender, age, body mass index (BMI), and vital status were all extracted from EMR, along with any previous history of cancer (yes/no) or known comorbidities (none/one or more comorbidities). Current and former smokers were combined into an “ever smoker” category and compared to those without any history of smoking (never smoker). Treatment was assessed and patients were grouped by those whose treatment included BCG (BCG group), those who did not receive BCG but received chemotherapy (chemotherapy group), and those who received neither chemotherapy nor BCG (no chemotherapy group). IFN-γ signatures were calculated for the tumor samples and the range of IFN-γ signatures was divided into tertiles for each UBC cohort. IFN-γ signatures in the highest tertile were then compared to the signatures in the lowest two tertiles in the analyses. Vital status was extracted from EMR, and for individuals who did not have a date of death listed in their EMR, obituaries were searched. For those patients with an obituary, the funeral date was used as the date of death. Individuals who did not have a date of death but did enter hospice care were recorded as having died one month from the day they entered hospice care. If no evidence of a date of death, funeral, or entry into hospice care could be found, individuals were censored at the date of their last appointment as noted in the EMR. Progression was defined as the change in a patient’s UBC tumor type from NMIBC to either MIBC or MBC, or from MIBC to MBC. MBC is a terminal diagnosis without the ability to progress. The dates of the patients’ last appointment without evidence of progression and histological confirmation of progression were recorded from the EMR. Patients were recorded as having progressed on the date of their histological confirmation of progression. Patients who had no evidence of progression were censored at the date of their last appointment (Fig 1).

### Tumor sample analysis

For the three cohorts, two de-identified curls with a thickness of 20 μm were cut and sent to AstraZeneca. RNA was extracted for sequencing. A database was developed to track the shipment of de-identified samples and the receipt of the results. Analyses were performed by AstraZeneca using Ion AmpliSeq sequencing technologies to determine the IFN-γ signature for each sample. The IFN-γ signature was calculated using a housekeeping gene expression read count as a baseline value. The IFN-γ signature value counts for each sample were subtracted from the baseline value. Values closer to zero indicated higher IFN-γ expression indicating that IFN-γ expression is reflective of the level of immune cell infiltration and activation.

### Statistical analysis

Descriptive statistics were performed for the demographic characteristics of the patients according to the IFN-γ expression status for the three different bladder cancer cohorts (signature in the highest tertile or the lowest two tertiles). In each of the UBC cohorts, univariate cox regression analyses were performed for OS of each variable. Multivariable cox regression models, adjusted for age, gender, BMI, history of cancer, comorbidities, and smoking status, were created for OS, based on data availability. Treatment type was also included in the model for the NMIBC cohort, but because BCG is not recommended for MIBC or MBC, treatment type was not included in the model for either of those cohorts. Kaplan-Meier survival plots showing OS were created for each cohort. PFS was assessed via Kaplan-Meier survival plots and log-rank testing for NMIBC and MIBC patients. PFS could not be calculated for MBC patients because MBC is a terminal diagnosis.

## Results

**Fig 2:**
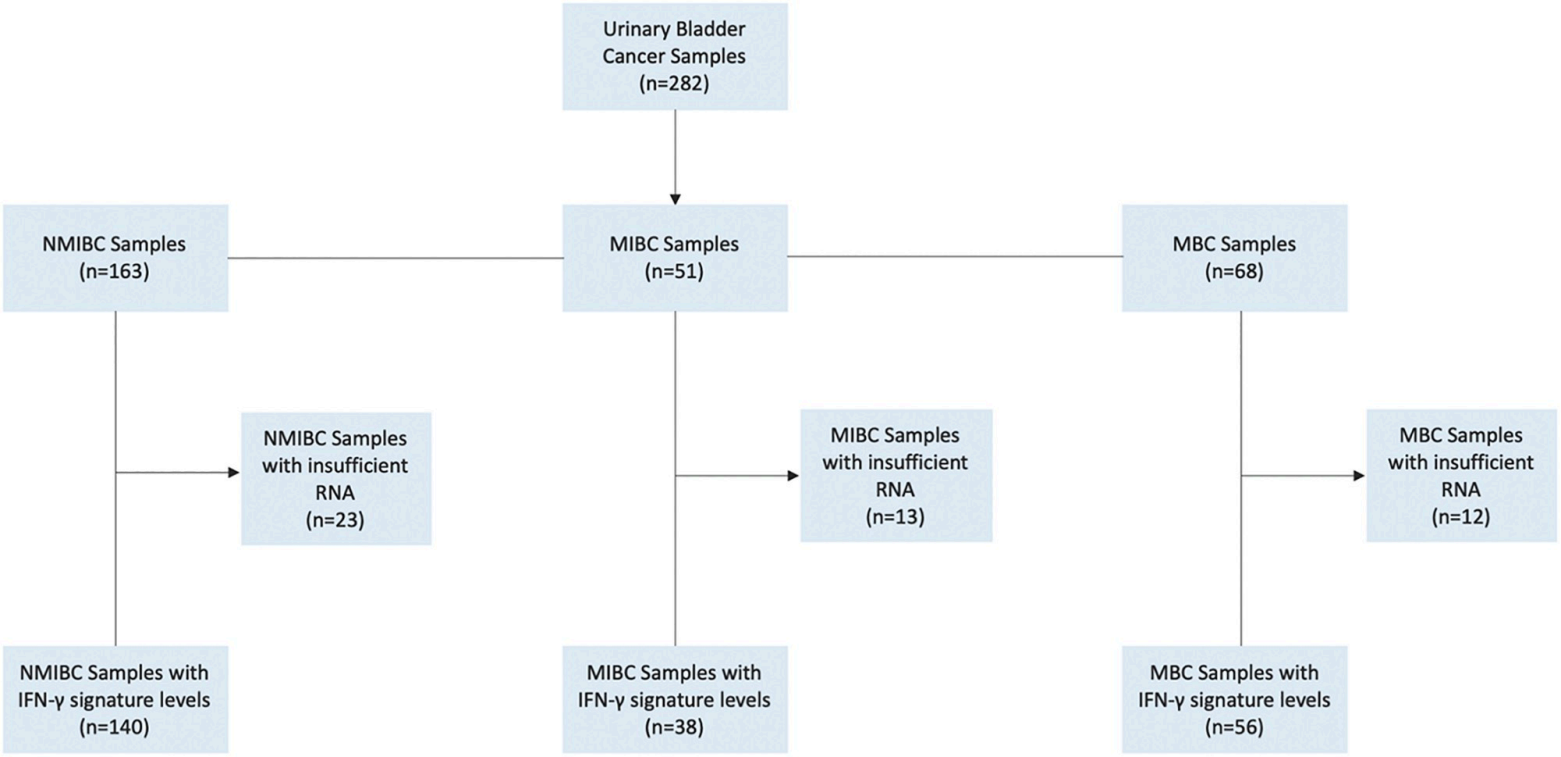
Sample flow diagram. Urinary bladder cancer study. Mt Sinai hospital system and North Shore Long Island Jewish health system, New York, New York. 2004–2017.

Among the 282 tumor samples collected, insufficient RNA was obtained from 48 samples to allow for IFN-γ signature determination. Therefore, IFN-γ signatures were calculated for the remaining 234 samples, consisting of 140 NMIBC samples, 38 MIBC samples, and 56 MBC samples for analysis (Fig 2). The samples were not longitudinally acquired (patients who transitioned from NMIBC → MIBC → MBC were not available) and there was no overlap between the NMBIC and the MIBC/MBC cohorts. Comparisons between those patients with measured IFN-γ and those without did not reveal any significant differences in patient characteristics (data not shown). Age, gender, and history of cancer were similar across the three UBC cohorts. Descriptive statistics are noted for all samples below and further detail is provided in Table 1 and S1 Table. The multivariable models for each UBC cohort were tested for interactions between time and each variable. None of the interaction variables were statistically significant so proportionality over time was assumed for each model.

**Table 1:** Characteristics of bladder cancer patients by stage. Mt Sinai Hospital system and North Shore Long Island Jewish Health System, New York, New York. 2004–2017.

	Non-muscle invasive bladder cancer, *n* (%)	Muscle invasive bladder cancer, *n* (%)	Metastatic bladder cancer, *n* (%)
	Overall (*N* = 140)	Overall (*N* = 38)	Overall (*N* = 56)
Gender			
Male	109 (77.9)	25 (65.8)	39 (69.6)
Female	31 (22.1)	13 (34.2)	17 (30.4)
Age (years)^a^, mean (SD)	70.8 (10.9)	73.5 (8.9)	70.0 (8.1)
BMI (kg/m^2^) ^a^, mean (SD)	27.6 (4.6)	27.1 (4.6)	27.0 (6.1)
History of cancer			
Yes	49 (35.0)	14 (36.8)	20 (35.7)
No	91(65.0)	23 (60.5)	36 (64.3)
Missing	0 (0.0)	1 (2.6)	0 (0.0)
Number of comorbidities			
0	78 (55.7)	14 (36.8)	34 (60.7)
1+	61 (43.6)	23 (60.5)	22 (39.3)
Missing	1 (0.7)	1 (2.6)	0 (0.0)
Smoking			
Never smoker	42 (30.0)	8 (21.0)	13 (23.2)
Ever smoker	92 (65.7)	29 (76.3)	32 (57.1)
Missing	6 (4.3)	1 (2.6)	11 (19.6)
Chemotherapy			
Any Chemotherapy	88 (62.9)	-	23 (41.1)
No chemotherapy	33 (23.6)	-	-
Missing	19 (13.6)	-	33 (58.9)
Tertile of INF- γ signature levels			
Cut off value for highest tertile	-4.62	-3.61	-2.98
Lowest tertiles	93 (66.4)	25 (65.8)	37 (66.1)
Highest tertile	47 (33.6)	13 (34.2)	19 (33.9)
IFN-γ signature, mean (SD)	-6.2 (2.5)	-4.3 (2.1)	-3.7 (1.4)
Vital status			
Alive	128 (91.4)	21 (55.3)	22 (39.3)
Dead	12 (8.6)	16 (42.1)	34 (60.7)
Missing	0 (0.0)	1 (2.6)	0 (0.0)
Follow-up time (months), median (range)	41.7 (1.8–153.5)	13.0 (0.7–64.4)	12.9 (0–89.8)

^a^One sample with an IFN-γ signature is missing all their demographic information except for gender including vital status.

### NMIBC

One hundred and forty individuals with NMIBC were identified and their tumor samples were evaluated for IFN-γ signatures. The majority of NMIBC individuals were men (78%) with a mean age of 71 years and mean BMI of 27.6 kg/m^2^. Most individuals did not have a history of cancer (65%) or other comorbidities (56%), although the majority did have a history of smoking (66%). Half of NMIBC individuals were treated with BCG (50%) while 13% of NMIBC individuals received other chemotherapy and about a quarter did not receive any chemotherapy. The median (range) follow-up time was 41.7 (1.8–153.5) months. There were 12 confirmed deaths in the NMIBC cohort with 128 individuals alive at the time of their last EMR entry. The mean ± standard deviation (SD) IFN-γ signature was -6.2 ± 2.5 and the cutoff for a signature in the highest tertile was -4.62 (Table 1). Of the 140 patients with NMIBC, 47 (34%) had a signature in the highest tertile (Table 1). Patients who received BCG therapy were significantly more likely to have an IFN-γ signature level in the highest tertile (*P* < 0.01, S1 Table).

**Fig 3:**
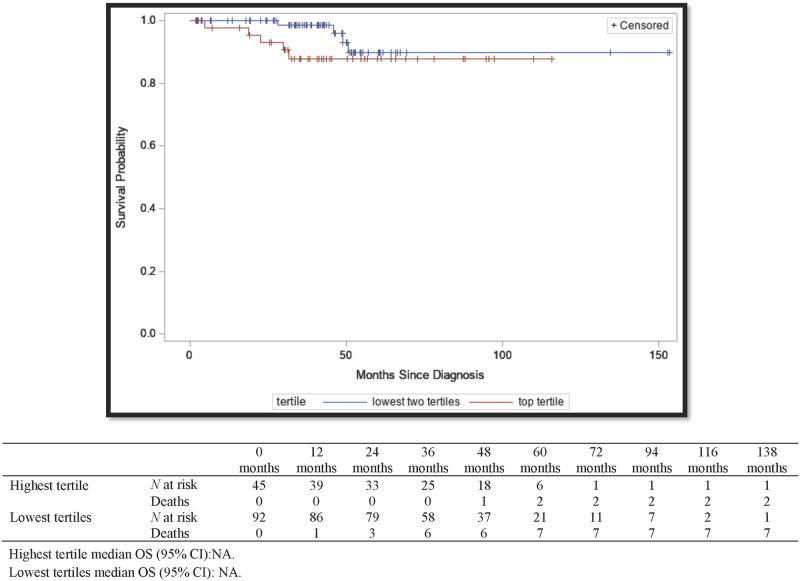
Kaplan–Meier survival curves for overall survival of NMIBC by highest tertile of IFN-γ signature vs the lowest two tertiles. Mt Sinai hospital system and North Shore Long Island Jewish health system, New York, New York. 2004–2017. Highest tertile median OS (95% CI):NA. Lowest tertiles median OS (95% CI): NA.

Median OS could not be calculated for patients with NMIBC, as most were alive at the end of the study (Fig 3). Regardless, there was no statistically significant difference between the two groups in survival at the end of the study period (*P* = 0.20). PFS could not be calculated for NMIBC patients because none of them progressed during the study period. Patients with IFN-γ signatures in the highest tertile were at decreased risk of mortality although the results were not statistically significant in the adjusted (adjusted for age, gender, BMI, history of cancer, comorbidities, smoking, and BCG chemotherapy) multivariable cox regression model (adjusted hazard ratio [aHR] = 0.73 [95% CI, 0.07–7.17]) (Table 2). There was one more patient for the analysis of OS than the multivariable cox regression because one patient was missing most of their data and they could not be included in the multivariable cox regression.

**Table 2:** Hazard ratios (HR) and 95% confidence intervals (CI) for the association between INF- γ signature levels and death among bladder cancer patients by stage. Mt Sinai hospital system and North Shore Long Island Jewish health system, New York, New York. 2004–2017.

	Non-muscle invasive bladder cancer		Muscle invasive bladder cancer		Metastatic bladder cancer	
	hazard ratio (95% CI)		hazard ratio (95% CI)		hazard ratio (95% CI)	
	Univariate	Multivariable model*	Univariate	Multivariable model*	Univariate	Multivariable model^a^
Tertile of INF- γ signature levels						
Lowest two tertiles combined	1.00 (reference)	1.00 (reference)	1.00 (reference)	1.00 (reference)	1.00 (reference)	1.00 (reference)
Highest tertile	0.34 (0.04–2.75)	0.73 (0.07–7.17)	0.11 (0.01–0.87)	0.09 (0.01–0.73)	0.65 (0.27–1.54)	0.85 (0.35–2.04)

^a^Adjusted for age, gender, BMI, history of cancer, comorbidities, and smoking status. BCG therapy was included in the model for NMIBC, but not for MIBC or MBC because it is not a recommended therapy for those diseases. Any chemotherapy use was included as a covariate in MBC models.

### MIBC

A total of 38 individuals with MIBC were identified and IFN-γ signatures were calculated from their tumors. The majority of MIBC individuals were men (66%) with a history of smoking (76%) and no history of cancer (61%). Individuals in this cohort were on average 74 years old with a BMI of 27.1 kg/m^2^. About 37% of these patients had no other comorbidities. The median (range) follow-up time was 13.0 (0.7–64.4) months and there were 16 confirmed deaths with 21 patients alive at the time of their last EMR entry. Subgroup analysis by grade could not be completed as all MIBC tumors available for study were grade 2. By stage, stage 2 tumors had higher IFN-γ expression compared to stages 3 and 4 (stage 2: -3.55 ± 1.86; stage 3: -4.09 ± 1.71; stage 4: -5.78 ± 2.33) (data not shown). The mean ± SD IFN-γ signature was –4.3 ± 2.1 and the cutoff for a signature in the highest tertile was –3.61 (Table 1). Of the 38 patients with MIBC, 13 (34%) had a signature in the highest tertile (Table 1).

**Fig 4:**
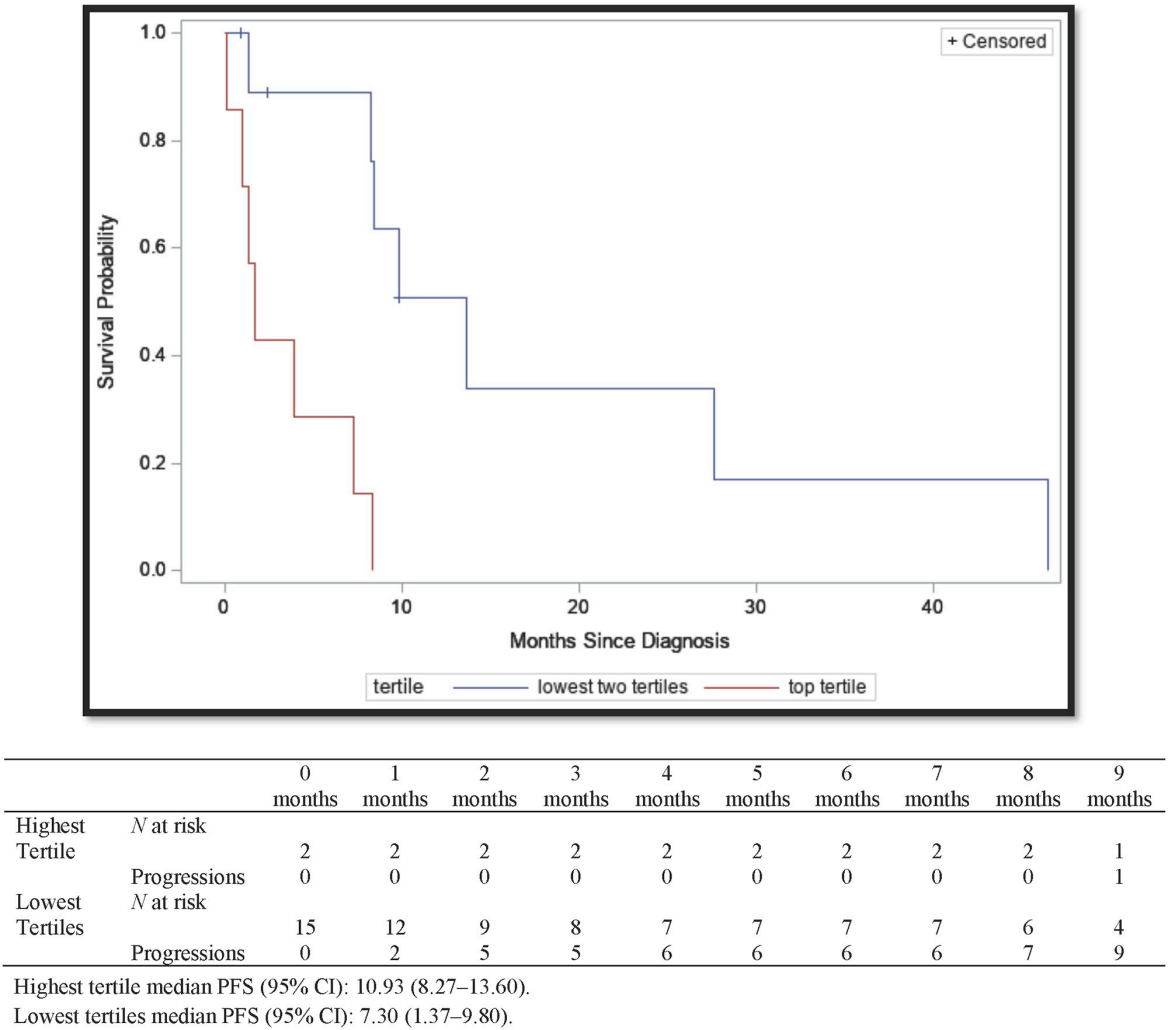
Kaplan-Meier survival curves for progression-free survival of MIBC by highest tertile of IFN-γ signature vs the lowest two tertiles. Mt Sinai hospital system and North Shore Long Island Jewish health system, New York, New York. 2004–2017. Highest tertile median PFS (95% CI): 10.93 (8.27–13.60). Lowest tertiles median PFS (95% CI): 7.30 (1.37–9.80).

**Fig 5:**
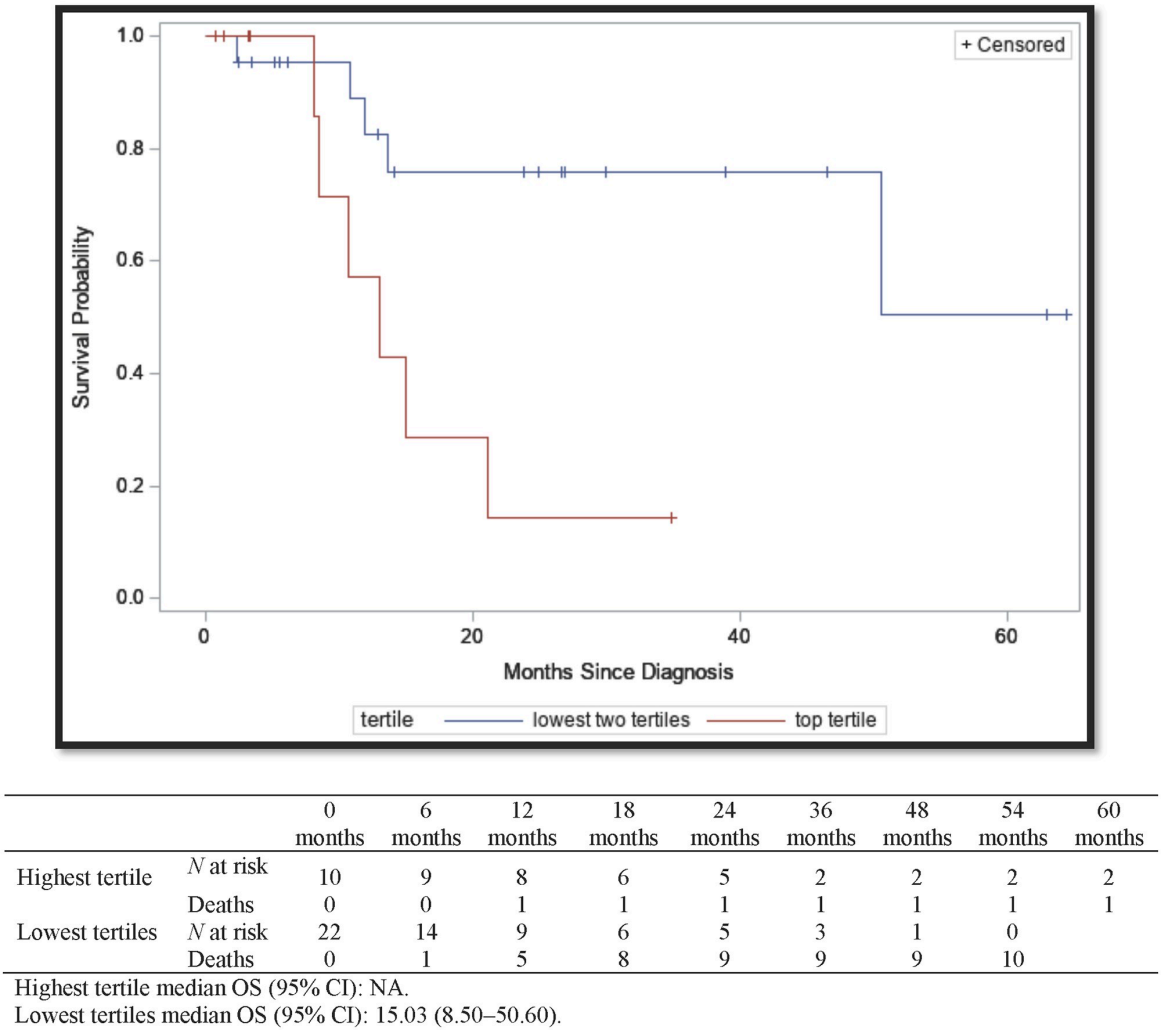
Kaplan-Meier survival curves for overall survival of MIBC by highest tertile of IFN-γ signature vs the lowest two tertiles. Mt Sinai Hospital System and North Shore Long Island Jewish Health System, New York, New York. 2004–2017. Highest tertile median OS (95% CI): NA. Lowest tertiles median OS (95% CI): 15.03 (8.50–50.60).

The median PFS time was significantly longer for patients with elevated IFN-γ signatures in the highest tertile at 10.93 (95% CI, 8.27–13.60) months when compared to 7.30 (95% CI, 1.37–9.80) months for those with IFN-γ signatures in the lowest tertiles (*P* < 0.01) (Fig 4). Only 11% of patients with an IFN-γ signature in the highest tertile died therefore OS could not be calculated, but the OS for those in the lowest tertiles was 15.03 (95% CI, 8.50–50.60) months (Fig 5) and the difference was statistically significant (*P* < 0.01). IFN-γ signatures in the highest tertile were associated with significantly decreased mortality in both the univariate (HR = 0.11 [95% CI, 0.01–0.87]) and adjusted multivariable cox regression model (aHR = 0.09 [95% CI, 0.01–0.73]) (Table 2).

### MBC

A total of 56 individuals with MBC were identified and IFN-γ signatures were calculated from their tumors. Most of them were male (70%) with a history of smoking (57%). On average, individuals were 70 years of age with a BMI of 27.0 kg/m^2^. Most individuals with MBC did not have a history of cancer (64%) or any comorbidities (61%). The median (range) follow-up time was 12.9 (0.0–89.8) months and there were 34 confirmed deaths with 22 patients alive at the time of their last EMR entry. Subgroup analysis by grade of MBC tumors showed higher IFN-γ expression in grade 2 tumors compared to grade 3 and higher (grade 2: -3.65 ± 1.30; grade 3+: -5.21 ± 2.60). By stage, stage 2 tumors had higher IFN-γ expression compared to stages 3 and 4 (stage 2: -2.80 ± 0.34; stage 3: -3.58 ± 1.37; stage 4: -5.78 ± 2.33). The mean ± SD IFN-γ signature was -3.7 ± 1.4 and the cutoff for a signature in the highest tertile was –2.98 (Table 1). Of the 56 patients with MBC, 19 (34%) had a signature in the highest tertile (Table 1).

**Fig 6:**
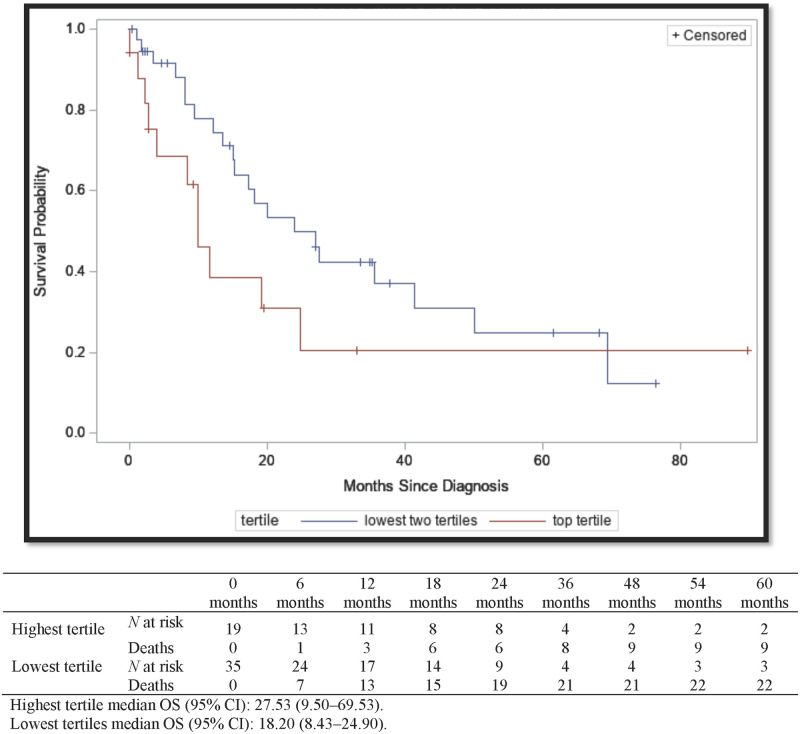
Kaplan-Meier survival curves for overall survival of MBC by highest tertile of IFN-γ signature vs. the lowest two tertiles. Mt Sinai hospital system and North Shore Long Island Jewish health system, New York, New York. 2004–2017. Highest tertile median OS (95% CI): 27.53 (9.50–69.53). Lowest tertiles median OS (95% CI): 18.20 (8.43–24.90).

The median OS (95% CI) for those with an IFN-γ signature in the lowest tertiles was shorter at 18.20 (95% CI, 8.43–24.90) months compared to 27.53 (95% CI, 9.50–69.53) months for patients with IFN-γ signatures in the highest tertile (Fig 6), although these differences were not statistically significant (*P* = 0.12). In the adjusted multivariable cox regression model, patients with IFN-γ signatures in the highest tertile were at a decreased risk of mortality (aHR = 0.85 [95% CI, 0.35–2.04]) compared to those in the lowest two tertiles, although the confidence intervals included “1” indicating that the results could be due to statistical chance (Table 2).

## Discussion

To our knowledge, this is the first real world study to examine the relation of IFN-γ and mortality in unselected cohorts of bladder cancer patients. The results of this study suggest that IFN-γ expression may be a predictor of mortality when viewed in conjunction with the extent of the disease, particularly in patients with MIBC.

The role of IFN-y in cancer occurrence and immunosurveillance is known. IFN-γ is produced in the early phases of immune reactions by several immune cells and signals the need for adaptive immunity. IFN-γ is implicated in driving monocytes, macrophages, and T cells to the site of inflammation and increases production of chemoattractants and sensitizes pro-apoptotic pathways, allowing for a greater immune response to tumor cells. In addition to enhancing immune response, IFN-γ acts directly on the tumor by reducing cell proliferation and hampering angiogenesis in tumor microenvironments, decreasing the ability of tumors to grow and receive nutrients [20].

Further evidence for the importance of IFN-γ in tumor suppression comes from preliminary data showing evidence that tumor infiltrating lymphocytes (TIL) in bladder cancer secrete IFN-γ. TIL, higher levels of which are associated with improved survival in MIBC, is sometimes used in immunotherapy with tumor cells extracted from patient tumors, proliferated in a lab, and subsequently reintroduced. In a 2018 study, researchers measured IFN-γ levels in TIL after co-culture with autologous tumor cells. The TIL was shown to have produced IFN-γ after co-culture with the autologous tumors, suggesting that IFN-γ was secreted in response to the exposure [21].

It also appears that IFN-γ is also one of the downstream mechanisms promoted by immunotherapy by intravesicular delivery of BCG, which is used to treat and prevent the recurrence of superficial bladder cancer. Although the exact mechanism by which BCG immunotherapy works is unclear, it has been suggested that the non-virulent mycobacterium bovis triggers a localized T helper 1 immune response, thereby increasing IFN-γ production and stimulating secretion of the chemokine IP-10, which helps to regulate inflammation. Research has shown that both IP-10 and IFN-γ levels in the urine of UBC patients who have received intravesicular BCG treatment increases overtime with the peak levels usually occurring between the fourth and sixth treatment [22]. This research suggests a link between receipt of BCG therapy and an increase in IFN-γ levels, which may then translate to increased survival.

There are several limitations to this study, which must be considered when interpreting these results. First, there was a limited number of available samples, particularly for the MIBC and MBC tumors, which was further exacerbated by the fact that nearly 20% of the samples generated insufficient nucleic acid for IFN-γ signature expression. This small sample size limited the ability to analyze several outcomes of interest including PFS in NMIBC patients and use of BCG in MIBC patients as there simply were not enough NMIBC patients who progressed or MIBC patients who received BCG therapy to calculate any meaningful statistics in this group. Second, because data was collected retrospectively from EMR, not all risk factors for bladder cancer were available to account for in the analyses. It is also possible that treatments which patients may have received outside of the Mount Sinai or Northwell Health Medical systems were not captured, leading to misclassification of treatment exposure.

The mean age of diagnosis for our cohort is consistent with SEER, an authoritative source for cancer statistics in the US, which shows individuals aged 65–74 years are most frequently diagnosed with UBC [23]. However, the number of comorbidities seen in our study compared to other early-stage bladder cancer populations differs. Megwalu et al. [24] found that about 35% of patients with non-invasive disease and 33% of patients treated with cystectomy did not have a comorbidity. Similarly, Strope et al. [25] found about 33% of early stage patients (stages 0 and 1) did not have a comorbidity. In contrast, we saw 56% of NMIBC, 37% of MIBC, and 61% of MBC patients reported no comorbidities, respectively, despite a mean age above 70 in each group. A study by Piccirillo et al. [26] of comorbidities in cancer patients found that by age 70, fewer than 30% of cancer patients reported no comorbidities. It is possible that patients had additional diagnosed comorbidities that were not included in their hospital EMR. Further, one of our requirements was that patients had to have a tissue sample available for IFN-γ analysis. Patients with tumor tissue may be more healthy than patients without as sicker patients may be less likely to undergo biopsy or surgical treatment for their tumor. These reasons may account for the differences we observed in 5-year survival rates in our sample compared to the SEER cancer cohort [23]. Five-year OS in our sample among NMIBC patients was 90.7% compared to 69.2% in localized bladder cancer patients in the SEER cancer cohort. Five-year OS in the MIBC sample was slightly higher at 44.3% compared to 36.5% among those with regional bladder cancer in SEER. Five-year OS in the MBC sample was 20.9% compared to 5.5% among those with distant bladder cancer in the SEER cohort [27].

Despite these limitations, this study has several strengths, primarily the long follow-up time of patients in the NMIBC cohort. Some of the patients in this study were followed for over 12 years, which provides ample time to establish OS. Additionally, the use of EMR rather than claims databases for patient and outcome identification allowed for better cohort identification and reduces misclassification. The use of EMR, obituary data, and the long follow-up period provided a more representative picture of the patient journey for people afflicted with UBC. Finally, comparisons between those patients with measured IFN-γ and those without did not reveal any significant differences in patient characteristics indicating that the IFN-γ group is not a biased group.

## Conclusions

This study suggests that IFN-γ expression in patients with UBC may be a prognostic factor of long-term patient outcomes. This is particularly true for those diagnosed with MIBC, where higher IFN-γ signature status was associated with a statistically significant increase in survival. Further research into the association between IFN-γ level and UBC mortality, as well as exploration of treatments that may raise IFN-γ level, is needed in well-defined and larger, unselected populations.

## Supporting information

S1 Table Characteristics of cancer patients by IFN-γ signature level. Mt Sinai hospital system and North Shore Long Island Jewish health system, New York, New York. 2004–2017.
(PDF)

## References

[1] Ploeg M, Aben KK, Kiemeney LA. The present and future burden of urinary bladder cancer in the world. World J Urol. 2009; 27(3):289–293. doi: 10.1007/s00345-009-0383-3 19219610 PMC2694323

[2] Edge S, Byrd DR, Compton CC, Fritz AG, Greene F, Trotti A. *AJCC cancer staging manual*. 8th ed. Chicago IL: American Joint Committee on Cancer, Springer; 2017.

[3] Babjuk M, Oosterlinck W, Sylvester R, Kaasinen E, Bӧhle A, Palou-Redorta J, et al. EAU guidelines on non-muscle-invasive urothelial carcinoma of the bladder, the 2011 update. Eur Urol. 2011; 59(6):997–1008. doi: 10.1016/j.eururo.2011.03.017 21458150

[4] Hall MC, Chang SS, Dalbagni G, Dalbagni G, Pruthi RS, Seigne JD, et al. Guideline for the management of nonmuscle invasive bladder cancer (stages Ta, T1, and Tis): 2007 update. J Urol. 2007; 178(6):2314–2330. doi: 10.1016/j.juro.2007.09.003 17993339

[5] Martin-Doyle W, Leow JJ, Orsola A, Chang SL, Bellmunt J. Improving selection criteria for early cystectomy in high-grade t1 bladder cancer: a meta-analysis of 15,215 patients. J Clin Oncol. 2015; 33(6):643–650. doi: 10.1200/JCO.2014.57.6967 25559810

[6] Sylvester RJ, van der Meijden APM, Lamm DL. Intravesical bacillus Calmette-Guerin reduces the risk of progression in patients with superficial bladder cancer: a meta-analysis of the published results of randomized clinical trials. J Urol. 2002; 168(5):1964–1970. 12394686 10.1016/S0022-5347(05)64273-5

[7] Winters BR, Wright JL, Holt SK, Dash A, Gore JL, Schade GR. Health related quality of life following radical cystectomy: comparative analysis from the Medicare Health Outcomes Survey. J Urol. 2018; 199(3):669–675. doi: 10.1016/j.juro.2017.08.111 28882404

[8] Morales A, Eidinger D, Bruce AW. Intracavitary Bacillus Calmette-Guerin in the treatment of superficial bladder tumors. J Urol. 1976; 116(2):180–183. doi: 10.1016/s0022-5347(17)58737-6 820877

[9] Ayers M, Lunceford J, Nebozhyn M, Murphy E, Loboda A, Albright A, et al. Relationship between immune gene signatures and clinical response to PD-1 blockade with pembrolizumab (MK-3475) in patients with advanced solid tumors. J Immunother Cancer. 2015; 3(Suppl 2):P80. doi: 10.1186/2051-1426-3-S2-P80

[10] Sundararajan S, Vogelzang NJ. Anti-PD-1 and PD-L1 therapy for bladder cancer: what is on the horizon? Future Oncol. 2015; 11(16):2299–2306. doi: 10.2217/fon.15.162 26260808

[11] Stewart R, Morrow M, Hammond SA, Mulgrew K, Marcus D, Poon E, et al. Identification and characterization of MEDI4736, an antagonistic anti-PD-L1 monoclonal antibody. Cancer Immunol Res. 2015; 3(9):1052–1062. doi: 10.1158/2326-6066.CIR-14-0191 25943534

[12] Expanding the reach of anti-PD-1 therapy. Cancer Discov. 2015; 5(7):684–685. doi: 10.1158/2159-8290.CD-NB2015-082 26034052

[13] Brahmer JR, Drake CG, Wollner I, Powderly JD, Picus J, Sharfman WH, et al. Phase I study of single-agent anti-programmed death-1 (MDX-1106) in refractory solid tumors: safety, clinical activity, pharmacodynamics, and immunologic correlates. J Clin Oncol. 2010; 28(19):3167–3175. doi: 10.1200/JCO.2009.26.7609 20516446 PMC4834717

[14] Chow LQM, Haddad R, Gupta S, Mahipal A, Mehra R, Tahara M, et al. antitumor activity of pembrolizumab in biomarker-unselected patients with recurrent and/or metastatic head and neck squamous cell carcinoma: results from the phase Ib KEYNOTE-012 expansion cohort. J Clin Oncol. 2016; 34(32):3838–3845. doi: 10.1200/JCO.2016.68.1478 27646946 PMC6804896

[15] Davidson M, Okines AF, Starling N. Current and future therapies for advanced gastric cancer. Clin Colorectal Cancer. 2015; 14(4):239–250. doi: 10.1016/j.clcc.2015.05.013 26524924

[16] Higgs BW, Morehouse C, Streicher K, Rebelatto MC, Steele K, Jin X, et al. Relationship of baseline tumoral IFNγ mRNA and PD-L1 protein expression to overall survival in durvalumab-treated NSCLC patients. J Clin Oncol. 2016; 34(15_suppl):Abstr 3036 doi: 10.1200/JCO.2016.34.15_suppl.3036

[17] Hino R, Kabashima K, Kato Y, Yagi H, Nakamura M, Honjo T, et al. Tumor cell expression of programmed cell death-1 ligand 1 is a prognostic factor for malignant melanoma. Cancer. 2010; 116(7):1757–1766. doi: 10.1002/cncr.24899 20143437

[18] Nakanishi J, Wada Y, Matsumoto K, Azuma M, Kikuchi K, Ueda S. Overexpression of B7-H1 (PD-L1) significantly associates with tumor grade and postoperative prognosis in human urothelial cancers. Cancer Immunol Immunother. 2007; 56(8):1173–1182. doi: 10.1007/s00262-006-0266-z 17186290 PMC11029839

[19] Rosenberg JE, Hoffman-Censits J, Powles T, van der Heijden MS, Balar AV, Necchi A, et al. Atezolizumab in patients with locally advanced and metastatic urothelial carcinoma who have progressed following treatment with platinum-based chemotherapy: a single-arm, multicentre, phase 2 trial. Lancet. 2016; 387(10031):1909–1920. doi: 10.1016/S0140-6736(16)00561-4 26952546 PMC5480242

[20] Kursunel MA, Esendagli G. The untold story of IFN-γ in cancer biology [published correction appears in Cytokine Growth Factor Rev. 2017; 35:97]. Cytokine Growth Factor Rev. 2016; 31:73–81. doi: 10.1016/j.cytogfr.2016.07.005 27502919

[21] Poch M, Hall M, Joerger A, Kodumudi K, Beatty M, Innamarato PP, et al. Expansion of tumor infiltrating lymphocytes (TIL) from bladder cancer. Oncoimmunology. 2018; 7(9):e1476816. doi: 10.1080/2162402X.2018.1476816 30228944 PMC6140546

[22] Paparo SR, Fallahi P. Bladder cancer and Th1 chemokines. Clin Ter. 2017; 168(1):e59–e63. doi: 10.7417/CT.2017.1984 28488840

[23] Surveillance, Epidemiology, and End Results (SEER) Program. Cancer Stat Facts: Bladder Cancer. National Cancer Institute, Bethesda, MD, USA. https://seer.cancer.gov/statfacts/html/urinb.html

[24] Megwalu II, Vlahiotis A, Radwan M, Piccirillo JF, Kibel AS. Prognostic impact of comorbidity in patients with bladder cancer. Eur Urol. 2008; 53(3):581–589. doi: 10.1016/j.eururo.2007.10.069 17997024 PMC2262100

[25] Strope SA, Ye Z, Hollingsworth JM, Hollenbeck BK. Patterns of care for early stage bladder cancer. Cancer. 2010; 116(11):2604–2611. doi: 10.1002/cncr.25007 20310051 PMC2876213

[26] Piccirillo JF, Vlahiotis A, Barrett LB, Flood KL, Spitznagel EL, Steyerberg EW. The changing prevalence of comorbidity across the age spectrum. Crit Rev Oncol Hematol. 2008; 67(2):124–132. doi: 10.1016/j.critrevonc.2008.01.013 18375141 PMC2536650

[27] SEER*Explorer: An interactive website for SEER cancer statistics. National Cancer Institute, Bethesda, MD, USA. https://seer.cancer.gov/explorer/

