# Supplementary material for: Interferon gamma expression and mortality in unselected cohorts of urothelial bladder cancer patients
Source: PLoS One. 2022 Aug 30;17(8):e0271339. doi: 10.1371/journal.pone.0271339 (PMC9426882; doi:10.1371/journal.pone.0271339)
Supplement: S1 Table — Mt Sinai hospital system and North Shore Long Island Jewish health system, New York, New York. 2004–2017. (PDF) [file pone.0271339.s001.pdf]

S1 Table. Characteristics of cancer patients by IFN-γ signature level. Mt Sinai hospital system and North Shore Long Island Jewish health system, New York, New York. 2004–2017.

	**Non-Muscle Invasive Bladder Cancer, *n*(%)**			**Muscle Invasive Bladder Cancer, *n* (%)**			**Metastatic bladder cancer, *n* (%)**		
	Highest tertile (*n*=47)	Lowest two tertiles (*n*=93)	Fisher's exact test *P*-value[b]	Highest tertile (*n*=13)	Lowest two tertiles (*n*=25)	Fisher's exact test *P*-value[b]	Highest tertile (*n*=19)	Lowest two tertiles (*n*=37)	Fisher's exact test *P*-value[b]
Gender			0.52			0.08			0.76
Male	35 (74.5)	74 (79.6)		6 (46.2)	19 (76.0)		14 (73.7)	25 (67.6)	
Female	12 (25.5)	19 (20.4)		7 (53.8)	6 (24.0)		5 (26.3)	12 (32.4)	
Age (years),[a] mean (SD)	73.1 (11.2)	69.6 (10.7)	0.07	70.3 (12.1)	75.0 (6.7)	<0.0001	68.7 (8.1)	70.6 (8.1)	0.41
BMI (kg/m^2),[a] mean (SD)	27.2 (5.0)	27.8 (4.4)	0.46	27.5 (3.7)	26.9 (5.0)	<0.0001	28.0 (8.4)	26.4 (4.5)	0.44
History of Cancer			0.23			0.09			0.77
Yes	18 (38.3)	31 (33.3)		4 (30.8)	10 (40.0)		6 (31.6)	14 (37.8)	
No	29 (61.7)	62 (66.7)		8 (61.5)	15 (60.0)		13 (68.4)	23 (62.2)	
Missing	0 (0.0)	0 (0.0)		1 (7.7)	0 (0.0)		0 (0.0)	0 (0.0)	
Number of Comorbidities			0.08			0.09			1.00
0	28 (59.6)	50 (53.8)		5 (38.5)	9 (36.0)		12 (63.2)	22 (59.5)	
1[b]	19 (40.4)	42 (45.2)		7 (53.8)	16 (64.0)		7 (36.8)	15 (40.5)	

Missing	0 (0.0)	1 (1.1)		1 (7.7)	0 (0.0)		0 (0.0)	0 (0.0)	
Smoking			0.50			0.48			0.74
Never smoker	17 (36.2)	25 (26.9)		3 (23.1)	5 (20.0)		4 (21.1)	9 (24.3)	
Ever smoker	28 (59.6)	64 (68.8)		9 (69.2)	20 (80.0)		10 (52.6)	22 (59.5)	
Missing	2 (4.3)	4 (4.3)		1 (7.7)	0 (0.0)		5 (26.3)	6 (16.2)	
Chemotherapy			<0.01			NA			NA
BCG	30 (63.8)	40 (43.0)		NA	NA		NA	NA	
Other chemotherapy	2 (4.3)	16 (17.2)		NA	NA		NA	NA	
No chemotherapy	9 (19.1)	24 (25.8)		NA	NA		NA	NA	
Missing	6 (12.8)	13 (14.0)		NA	NA		NA	NA	
Vital Status			0.30			0.08			0.40
Alive	43 (91.5)	85 (91.4)		9 (69.2)	12 (48.0)		9 (47.4)	13 (35.1)	
Dead	4 (8.5)	8 (8.6)		3 (23.1)	13 (52.0)		10 (52.6)	24 (64.9)	
Missing	0 (0.0)	0 (0.0)		1 (7.7)	0 (0.0)		0 (0.0)	0 (0.0)	
Follow-up time (months),[a] median (range)	41.7 (2.2–152.8)	41.8 (1.8–153.5)	0.16	24.4 (3.5–64.4)	9.6 (0.7–50.6)	<0.0001	15.23 (1.0–69.5)	11.6 (0.0–89.8)	0.63
IFN- **γ** signature, mean (SD)	NA	NA		NA	NA		NA	NA	

NA: Not available.

[a]Student's T-test. [b]Measuring if there is a statistically significant difference between the values in the highest tertile group and the values in the lowest two tertiles group for each variable.
